# Geographical information system and environmental epidemiology: a cross-sectional spatial analysis of the effects of traffic-related air pollution on population respiratory health

**DOI:** 10.1186/1476-069X-10-12

**Published:** 2011-03-01

**Authors:** Daniela Nuvolone, Roberto della Maggiore, Sara Maio, Roberto Fresco, Sandra Baldacci, Laura Carrozzi, Francesco Pistelli, Giovanni Viegi

**Affiliations:** 1 Epidemiology Unit, Regional Agency of Public Health of Tuscany (ARS), Via Pietro Dazzi 1, I-50141 Florence, Italy; 2 Information Systems Technology Centre, Institute of Information Science and Technologies 'Alessandro Faedo', Italian National Research Council (ISTI-CNR), Via G. Moruzzi 1, I-56124 Pisa, Italy; 3 Pulmonary Environmental Epidemiology Unit, Institute of Clinical Physiology, Italian National Research Council (IFC-CNR), Via Trieste 41, I-56126 Pisa, Italy; 4 Institute of Biomedicine and Molecular Immunology, Italian National Research Council (IBIM-CNR), Via Ugo La Malfa 153, I-90146 Palermo, Italy

## Abstract

**Background:**

Traffic-related air pollution is a potential risk factor for human respiratory health. A Geographical Information System (GIS) approach was used to examine whether distance from a main road (the Tosco-Romagnola road) affected respiratory health status.

**Methods:**

We used data collected during an epidemiological survey performed in the Pisa-Cascina area (central Italy) in the period 1991-93. A total of 2841 subjects participated in the survey and filled out a standardized questionnaire on health status, socio-demographic information, and personal habits. A variable proportion of subjects performed lung function and allergy tests. Highly exposed subjects were defined as those living within 100 m of the main road, moderately exposed as those living between 100 and 250 m from the road, and unexposed as those living between 250 and 800 m from the road. Statistical analyses were conducted to compare the risks for respiratory symptoms and diseases between exposed and unexposed. All analyses were stratified by gender.

**Results:**

The study comprised 2062 subjects: mean age was 45.9 years for men and 48.9 years for women. Compared to subjects living between 250 m and 800 m from the main road, subjects living within 100 m of the main road had increased adjusted risks for persistent wheeze (OR = 1.76, 95% CI = 1.08-2.87), COPD diagnosis (OR = 1.80, 95% CI = 1.03-3.08), and reduced FEV_1_/FVC ratio (OR = 2.07, 95% CI = 1.11-3.87) among males, and for dyspnea (OR = 1.61, 95% CI = 1.13-2.27), positivity to skin prick test (OR = 1.83, 95% CI = 1.11-3.00), asthma diagnosis (OR = 1.68, 95% CI = 0.97-2.88) and attacks of shortness of breath with wheeze (OR = 1.67, 95% CI = 0.98-2.84) among females.

**Conclusion:**

This study points out the potential effects of traffic-related air pollution on respiratory health status, including lung function impairment. It also highlights the added value of GIS in environmental health research.

## Background

In recent years, despite significant improvements in fuel and engine technology, emissions from traffic have become a major source of air pollution, mainly in urban areas. In Europe, exhaust from motor vehicle traffic is considered to be the most significant source of nitrogen oxides (NO_x_), carbon monoxide (CO) and non-methane volatile organic compounds (NMVOCs), as well as the second most important source of particulate emissions [1].

Studies of long-term exposure to air pollution suggest an increased risk of chronic respiratory illnesses [2-4]. Short-term exposures to high concentrations have been associated with higher prevalence rates of bronchitis, asthma and respiratory symptoms [5-8].

In urban environments, mainly in areas where population and traffic density are relatively high, human exposure to hazardous substances is expected to be relevant. This is often the case near busy traffic axes in city centers where urban topography and microclimate may contribute to poor dispersion conditions. There is growing epidemiological evidence of increased frequency of respiratory symptoms among people living close to major roads [9-12]. Few studies have focused on the sex-specific associations between exposure to urban air pollution and respiratory health [13].

Advances in Geographical Information System (GIS) technology, along with the increasing availability of geographical data, have provided new opportunities for environmental epidemiologists to study associations between environmental exposure and spatial distribution of disease. GIS permits spatial linking of different types of data (e.g., residential addresses, environmental exposure levels, demographic information), as well as automated address matching, buffer analysis, spatial query and polygon overlay analysis [14,15].

A number of methods for exposure assessment have been developed. Researchers have generally used self-reported or measured traffic density, and self-reported or measured distance of the home from the nearest street [16-19]. Other teams have used both sets of information, traffic density and distance [20-23]. Traffic air pollutant dispersion models and land use regression (LUR) models have been developed to improve the estimation of exposure levels [15,24-26], and they have sometimes been used in epidemiology [14,27,28].

The aim of our study was to evaluate the sex-specific associations between living near a main road (assessed through a GIS-based methodology) and respiratory health status in a general population sample.

## Methods

### Setting and study population

Since 1980 the Pulmonary Environmental Epidemiology Unit of the Institute of Clinical Physiology of the Italian National Research Council (CNR) has performed epidemiological surveys to assess the effects of outdoor [29-32] and indoor [33,34] air pollution on human health.

**Figure 1:**
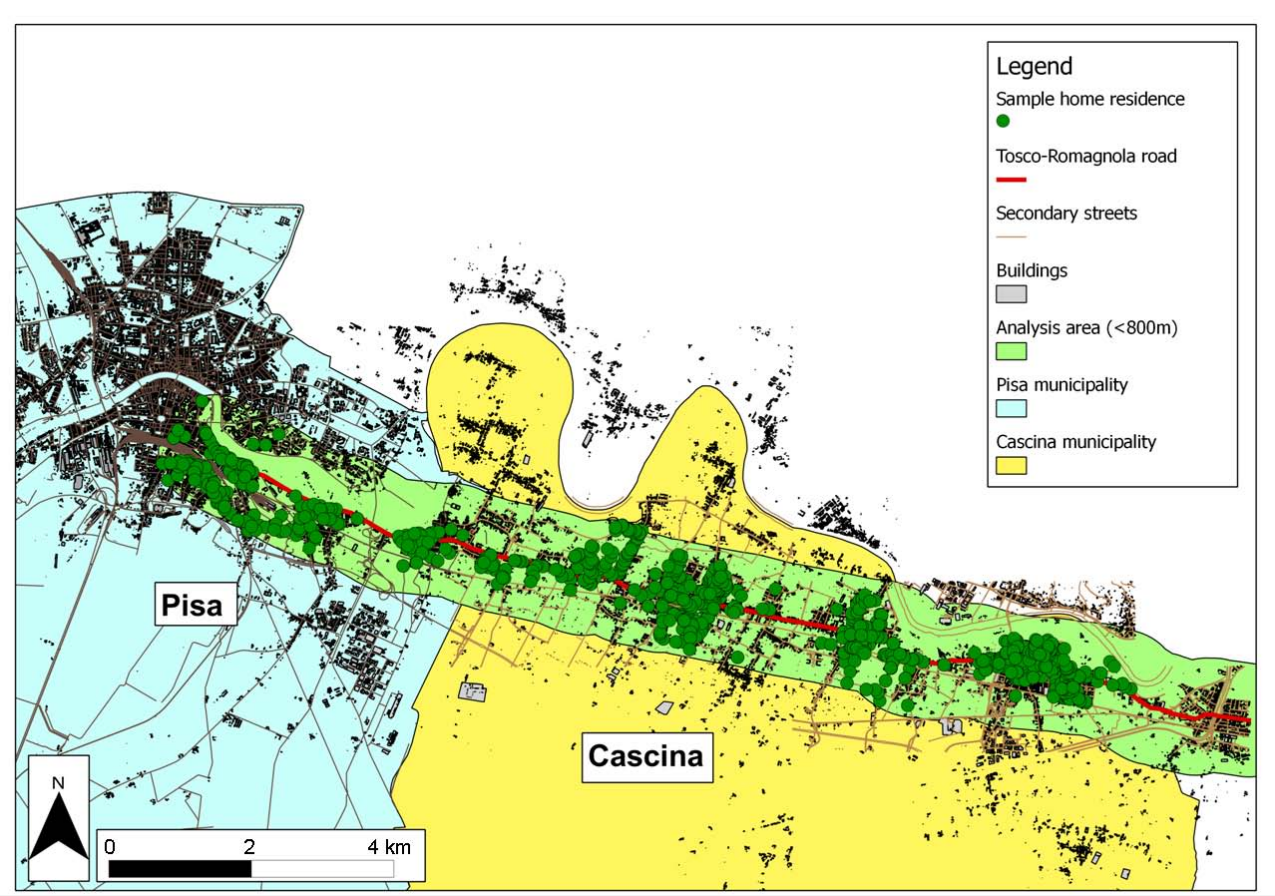
**Study area and geocoding of population sample**. Map representing subjects geocoding in their own home residence address, along the major road, the Tosco-Romagnola road. Each dot on the map represents each subject's home. The map indicates the road and railway network and buildings in the two municipalities.

From 1991 to 1993 a survey was conducted in the area of Pisa (Tuscany), along a main road, called the Tosco-Romagnola road, connecting Pisa to Florence and characterized by high traffic volume (mean daily values ~14700 vehicles, measured during the hours 07.00-21.00). Figure 1 shows a map of the study area, representing the boundaries of the two municipalities involved (Pisa and Cascina), the main road, and the secondary streets. As shown in the map, the central and eastern side of the Tosco-Romagnola road had typical characteristics of a suburban/rural area with sparse buildings and intersections with very small streets, suggesting a major role of the Tosco-Romagnola road in air pollutant emissions. The last western part of the Tosco-Romagnola road, which enters the urban area of Pisa, has different characteristics with regard to other main roads, other air pollution sources, and other types of buildings.

Annual concentrations of total suspended particulates (TSP) were provided by the Pisa Province Unit of the Environmental Protection Agency, along with integrated measurements of sulfur dioxide (SO_2_): annual means were 24 μg/m^3 ^for SO_2 _and 99 μg/m^3 ^for TSP, for the entire area around the main road.

Subjects participating in the survey (n = 2841) were sampled using a multistage stratified family-cluster design. They were investigated with a protocol including: the CNR questionnaire on respiratory symptoms/diseases and risk factors, lung function tests, skin prick tests, and blood samples for immunoglobulin E (IgE) determination [35].

### Exposure assessment

Subjects were integrated in a Geographical Information System. Geocoding was done using home residence addresses. For the subjects geocoding, we used cartographic data provided by the GIS Service of Pisa and Cascina municipalities: buildings, streets, topography, population addresses, and house numbers. We applied addresses geocoding techniques provided by ArcMap 8.2 (ESRI): a file extracted from the epidemiological questionnaire containing participants' addresses (street names and house numbers) was matched with vector data. Cartographic data provided by Pisa and Cascina municipalities contained the exact location of house numbers, as in real life; a direct inspection was performed in case of ambiguity or uncertainty. Each subject is shown on a map as a precise mark corresponding to his/her home address, identified by street name and house number.

As described in the previous section, in order to minimize the effects of other air pollution sources (industries, other main roads), mainly on the last western part of the Tosco-Romagnola road, only subjects living within 800 m of the main road (n = 2062, i.e. 73% of the total sample) were selected: this cut-off permitted us to also exclude the more rural area of the central-eastern side, characterized by a different kind of buildings (villas, isolated houses) and by different socio-economic status (Figure 1). It is important to underline that for the central and eastern part of the road more than 90% of our sample lived within 800 m of the road.

**Figure 2:**
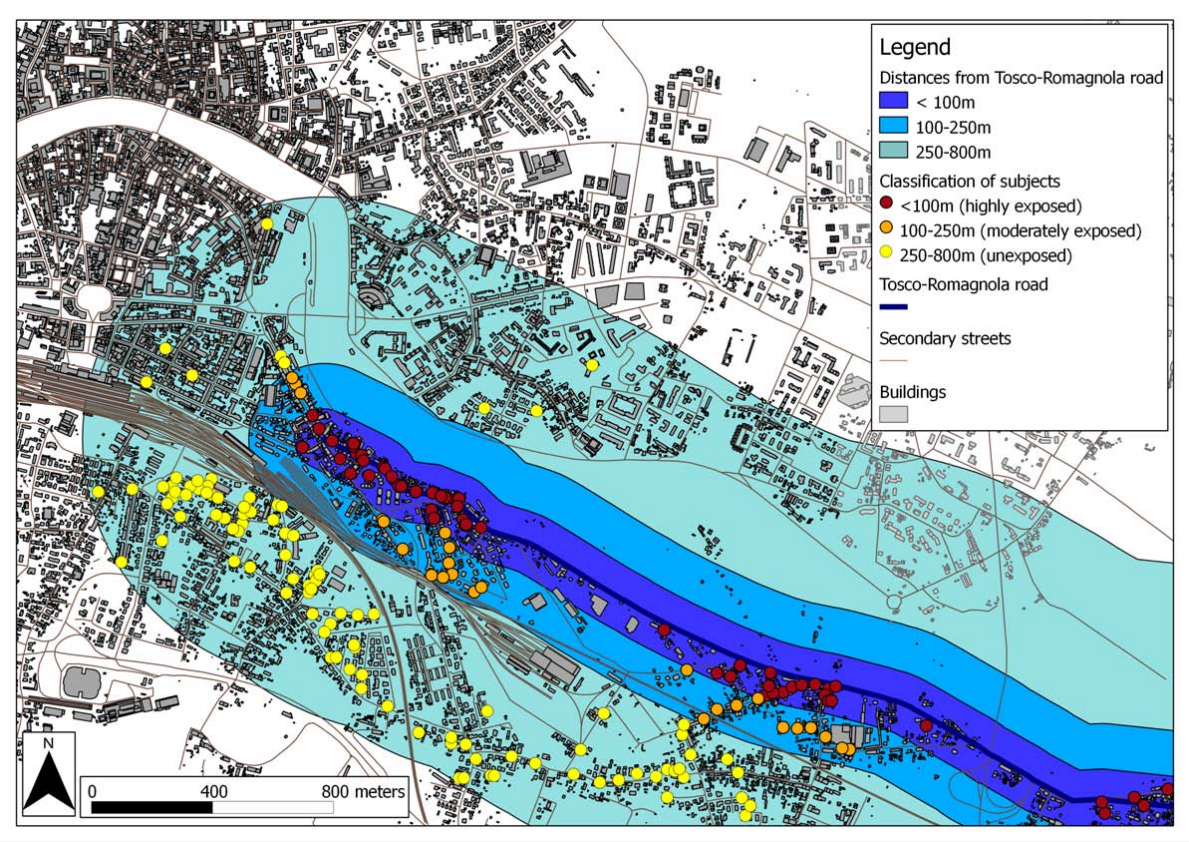
**Classification of subjects based on the distance of each home from the main road**. Zoomed map representing the classification of subjects according to the distance of each home from the main road. Highly exposed subjects are those living in the buffer area 0-100 m from the road, moderately exposed subjects living in the buffer area 100-250 m and unexposed are those living between 250 and 800 m from the road.

Distances of houses from the main road (the Tosco-Romagnola road) were used to assess traffic-related pollution exposure. Using GIS buffering and overlaying functionalities, we classified the population sample in three groups (see Figure 2): highly exposed (people living within 100 m of the main road), moderately exposed (people living between 100 m and 250 m from the main road) and unexposed subjects (people living between 250 m and 800 m from the main road). These cut-off values were selected based on the results of previous studies showing increased exposure and risk of respiratory symptoms within short distances from the roads [9-13,23].

### Health outcomes assessment

Subjects filled out a CNR standardized interviewer-administered questionnaire. This was the Italian version of the National Heart Blood and Lung Institute (NHBLI, USA) questionnaire including more than 70 questions on demographic aspects, general health status, lifestyle, potential risk factors (smoking habits, passive smoking exposure, occupational exposure, indoor exposure) [30].

The following respiratory symptoms/diseases were considered for the analyses:

• chronic cough (or phlegm): cough (or phlegm) apart from common colds for at least three months of the year for at least two years

• attacks of shortness of breath with wheeze: any attack of shortness of breath with wheeze, apart from common colds

• persistent wheeze: wheeze, for at least six months of the year, apart from common colds

• dyspnea I+ grade: shortness of breath when hurrying on level ground or walking up a slight hill (I grade dyspnea) or when walking on level ground with persons of the same age (II+ grade dyspnea)

• chronic obstructive pulmonary disease (COPD): reported diagnosis of emphysema or chronic bronchitis

• allergy symptoms: hay fever or any other condition making the nose runny or stuffy, apart from common colds, eye redness, itching, burning and eczema

• asthma: reported diagnosis.

In addition, the investigated subjects performed skin-prick tests for common airborne allergens, serum IgE determination, lung function tests and nonspecific bronchial challenge test with methacholine.

A skin-prick test result was considered positive if it yielded at least one wheal with a mean diameter of at least 3 mm (skin test_3 mm pos) or 5 mm (skin test_5 mm pos), after subtracting the diameter of the negative control reaction.

Total serum immunoglobulin E (IgE) was measured and transformed in logarithm10 values (IgE_log) to obtain a normal distribution.

Lung function tests were carried out: slow vital capacity, CO single breath diffusing capacity (DLCO) and forced expirograms. Values for spirometry parameters were all expressed in % predicted [36], with the exception of the ratio between forced expiratory volume in the first second and forced vital capacity (FEV_1_/FVC) and of the ratio between FEV_1 _and vital capacity (FEV_1_/VC), which were expressed in percentage of observed values.

The results of the non-specific bronchial challenge test with methacholine were expressed using a continuous variable to characterize bronchial reactivity, the slope of the dose-response curve; the slope was transformed using the natural logarithm (slope_ln) because the data distribution was highly skewed, and a small constant (+ 2.57) was added to allow logarithmic transformation of negative and zero values.

A variable proportion of subjects agreed to perform these tests. Skin prick tests were performed for 1608 subjects (78%); serum IgE determination for 1409 subjects (68%); lung function tests for 1402 (68%); and bronchial responsiveness to methacholine challenge for 859 (42%). Subjects involved in lung function and allergy tests, compared to those not involved, were more likely men, smokers, young/adults, exposed to passive smoking and with high education levels (data not shown).

### Potential confounders

The following potential confounders, collected through questionnaire, were considered:

• age groups: < 25, 25-64, > 64 years. These groups were chosen in order to make possible comparisons with our previous studies [29]. Moreover, these cut-offs allow us to identify the most susceptible categories, i.e. the young people and the elderly

• smoking habits: non-smokers (those who had never smoked any kind of tobacco regularly); smokers (those who currently smoked at least one cigarette daily); ex-smokers (those who had smoked regularly in the past until six months or more before the examination, but did not smoke at the moment of the examination)

• passive smoking exposure: exposure to the smoke from other people

• educational level: low (no education/primary school); medium (secondary school); high (high school/university)

• work position: manager/white collar, blue collar/farmer, merchant/craftsman and unemployed

• occupational exposure: exposure to fumes, gases, dust or chemicals in working environments

• number of hours spent at home (home residence exposure): more than or equal to 15 h; less than 15 h

• time of residence: more than or equal to five years; less than five years

• type of self-reported environmental exposure: traffic; other exposure.

### Statistical analysis

Chi-square test was used to compare symptoms/diseases prevalence rates between exposed and unexposed subjects regarding traffic-related pollution exposure. Separate analyses were performed for both sexes.

Objective test variables were analyzed either as continuous or categorical variables. Comparison of adjusted mean values of functional and allergologic parameters (IgE determination, bronchial reactivity and spirometry) among the three exposure classes was performed by analysis of variance (ANOVA). For lung function tests, the mean values were adjusted for the effects of age and smoking habits; for bronchial reactivity parameters, mean values were adjusted for age, smoking habits and predicted FEV_1_%. Post hoc test (Tukey test) was applied to perform all pair-wise comparisons of the ANOVA results in order to identify which means were significantly different from the others.

In addition, continuous variables were dichotomized and analyzed by chi-square: IgE and bronchial reactivity results were dichotomized through the 75^th ^percentile: 1.83 for the logarithm of IgE (IgE_log) and 2.22 for the logarithm of the slope of the dose-response bronchial reactivity curve (slope_ln); airway obstruction was defined as having an observed FEV_1_/FVC% less than 70%.

We applied multiple logistic regression models to assess the association between health outcomes and traffic-related pollution exposure taking into account the role of the independent risk factors. Odds ratios (OR) were stratified by sex and adjusted for the effects of age, education, smoking, passive smoking exposure, occupational exposure, working position, number of hours spent at home and time of residence.

A p-value less than 0.05 was considered statistically significant.

## Results

The study included 2062 subjects: mean age was 45.9 years for men (range 8-93 years) and 48.9 years for women (range 8-97 years). Children (0-14 years) comprised 5% of the study sample. General characteristics of the population sample are reported in Table 1, for men and women. A different distribution of potential confounding factors was observed between genders: females were older than males, current and previous smoking was more frequent in males than in females. Men had a higher education level and socio-economic status, but also a higher frequency of occupational exposure. Women tended to spend more time at home than men, as well as to perceiving vehicular traffic in the street of residence more frequently. Over 85% of the population had been living in the same house for more than five years.

**Table 1:** General characteristics of the population sample by gender.

	Males (n = 944)	Females (n = 1118)	p-value*
Age (years)	%	%	
8-24	20.4	16.4	
25-64	58.8	58.3	p = 0.010
65-97	20.8	25.3	
			
Smoking habits			
smokers	28.6	17.2	
ex-smokers	40.6	16.8	p < 0.001
non-smokers	30.8	66.0	
			
Passive smoking exposure			
yes	58.3	48.3	p < 0.001
no	41.7	51.7	
			
Educational level			
low	42.8	59.6	
medium	30.7	20.0	p < 0.001
high	26.5	20.4	
			
Work position			
manager/white collar	17.3	13.3	
blue collar/farmer	19.9	8.3	p < 0.001
merchant/craftsman	14.6	8.2	
unemployed	48.2	70.2	
			
Occupational exposure			
yes	58.1	28.7	p < 0.001
no	41.9	71.3	
			
Number of hours at home			
≥ 15 h	39.3	71.5	p < 0.001
< 15 h	60.7	28.5	
			
Time of residence			
< 5 years	11.8	10.4	p = 0.308
≥ 5 years	88.2	89.6	
			
Type of self-reported environmental exposure			
traffic	79.6	83.9	p = 0.076
other	20.4	16.1	

*p-values from Pearson chi-square test

The average (± standard deviation) distance of subjects' residences from the main road was 239 ± 189 m (median 200 m; minimum 1.5 m; maximum 785 m). Table 2 reports general characteristics of the population when stratified by the three distance classes and by sex. Among females, the elderly tended to live closer to the main road than younger people; females living within 100 m of the road tended to have lower socio-economic status and less passive smoking exposure and occupational exposure than females living farther away. Variables about self-reported perception of environmental exposure were correlated to distance classes used to define traffic-related exposure in both males and females, with subjects living within 100 m from the main road showing the highest self-reported exposure to traffic.

**Table 2:** Distribution of confounding factors by the distance classes in males and females.

	Males(n = 944)			Females(n = 1118)		
	Distance of residence to main road			Distance of residence to main road		
	<100 m	100-250 m	250-800 m	<100 m	100-250 m	250-800 m
	n = 263M	n = 279	n = 402	n = 322	n = 332	n = 464
	%	%	%	%	%	%
Age (years)						
8-24	19.4	22.6	19.7	13.4*	16.9	18.1
25-64	56.7	60.9	58.7	55.5	61.4	58.0
65-97	19.9	16.5	21.6	31.1	21.7	23.9
						
Smoking habits						
smokers	27.3	26.9	30.6	17.7	17.5	16.6
ex-smokers	43.4	39.8	39.3	17.7	16.2	16.6
non-smokers	29.3	33.3	30.1	64.6	66.3	66.8
						
Passive smoking exposure						
yes	59.1	57.7	58.2	43.2 #	49.1	51.3
no	40.9	42.3	41.8	56.8	50.9	48.7
						
Educational level						
low	41.1	45.2	42.3	65.5 #	57.8	56.9
medium	30.4	31.9	30.1	14.6	21.4	22.6
high	28.5	22.9	27.6	19.9	20.8	20.5
						
Work position						
manager/white collar	13.7	17.9	19.2	8.9 *	16.7	13.7
blue collar/farmer	19.5	19.7	20.3	10.1	6.1	8.7
merchant/craftsman	17.6	15.4	12.0	9.5	6.4	8.7
unemployed	49.2	47.0	48.5	71.5	70.8	68.9
						
Occupational exposure						
yes	57.0	59.5	57.7	26.4 #	25.9	32.3
no	43.0	40.5	42.3	73.6	74.1	67.7
						
Number of hours at home						
≥ 15 h	41.1	37.6	39.3	74.2	71.1	69.8
< 15 h	58.9	62.4	60.7	25.8	28.9	30.2
						
Time of residence						
< 5 years	14.2	10.8	11.0	11.6	8.1	11.2
≥ 5 years	85.8	89.2	89.0	88.4	91.9	88.8
						
Type of self-reported environmental contamination						
traffic	92.4***	65.2	75.2	93.6 ***	74.8	78.5
other	7.6	34.8	24.8	6.4	25.2	21.5

*** p < 0.001, ** p < 0.01, * p < 0.05, # 0.05 < p < 0.1 (borderline) from Pearson chi-square test; comparison between subjects living at different distances from the main road, separately in males and in females

As regards symptoms/diseases, persistent wheeze and COPD showed significantly higher prevalence rates in males living within 100 m of the main road; attacks of shortness of breath with wheeze, dyspnea and asthma showed significantly higher prevalence rates in females living within 100 m of the main road (Table 3).

**Table 3:** Prevalence rates of symptoms/diseases by the distance classes in males and females.

	Males			Females		
	Distance of residence to main road			Distance of residence to main road		
	<100 m	100-250 m	250-800 m	<100 m	100-250 m	250-800 m
	n = 263	n = 279	n = 402	n = 322	n = 332	n = 464
	%	%	%	%	%	%
Chronic cough	18.6	17.2	17.9	9.0	11.1	8.8
Chronic phlegm	22.8	17.6	21.1	7.8	7.2	5.8
Persistent wheeze	15.2 #	13.6	9.7	8.7	5.4	6.9
Dyspnea	17.9	16.1	18.4	35.4 **	28.3	23.9
COPD	14.4 #	9.7	9.2	4.3	2.4	2.6
Hay fever	16.7	21.1	19.2	18.3	16.3	22.0
Eye redness	17.1	17.6	19.2	22.7	23.8	22.5
Asthma	8.7	8.2	5.5	9.6 *	3.9	6.3
Eczema	10.3	7.9	9.2	11.2	12.3	12.9
Attacks of shortness of breath with wheeze	11.4	11.1	9.2	9.3 **	3.3	5.8

*** p < 0.001, ** p < 0.01, * p < 0.05, # 0.05 < p < 0.1 (borderline) from Pearson chi-square test; comparison between subjects living at different distances from the main road, separately in males and in females

Results of the comparison of adjusted mean values of functional and allergologic parameters among the exposure classes stratified by sex are reported in Table 4. Significantly lower FEV_1_/VC% and FEV_1_/FVC% values were observed in exposed males. The Tukey test highlighted that all significant p-values were associated with differences between subjects living within 100 m of the road (the highly exposed class) and subjects living between 250 m and 800 m from the road (unexposed). There were no significant differences among groups for the logarithm of serum IgE values, nor for the logarithm of the slope of the bronchial reactivity dose-response curve, though for the latter there was a trend between the three exposure classes.

**Table 4:** Comparison of adjusted mean values of functional and allergologic parameters by the distance classes in males and females.

	Males			Females		
	Distance of residence to main road			Distance of residence to main road		
	<100 m	100-250 m	250-800 m	<100 m	100-250 m	250-800 m
FEV_1_%^§^	98.67 (n = 191)	96.76 (n = 217)	98.44 (n = 303)	99.67 (n = 183)	100.38 (n = 214)	98.13 (n = 294)
FVC%^§^	101.02 (n = 191)	99.69 (n = 217)	99.06 (n = 303)	102.14 (n = 183)	101.31 (n = 214)	99.88 (n = 294)
FEV_1_/FVC%^§^	78.39 ** (n = 191)	78.49 (n = 217)	79.82 (n = 303)	80.43 (n = 183)	82.36 (n = 214)	81.43 (n = 294)
FEV_1_/VC%^§^	78.59 ** (n = 191)	78.33 (n = 217)	79.84 (n = 302)	80.69 (n = 183)	82.39 (n = 214)	81.80 (n = 294)
DLCO%^§^	77.50 (n = 156)	78.87 (n = 170)	77.33 (n = 245)	74.56 (n = 141)	76.62 (n = 160)	74.87 (n = 229)
IgE_log	1.63 (n = 191)	1.62 (n = 206)	1.54 (n = 291)	1.40 (n = 205)	1.33 (n = 208)	1.35 (n = 308)
Bronchial reactivity slope_ln	1.84 (n = 129)	1.77 (n = 148)	1.75 (n = 203)	2.05 (n = 87)	1.99 (n = 114)	1.98 (n = 157)

^§ ^Values are expressed in % predicted, with the exception of FEV_1_/FVC% and FEV_1_/VC% which are expressed in % observed

*** p < 0.001, ** p < 0.01, * p < 0.05, # 0.05 < p < 0.1 (borderline) by analyses of variance; comparison between subjects living at different distances from the main road, separately in males and in females

For all parameters mean values are adjusted for the effects of age and smoking habits, with the exception of the bronchial reactivity parameter where mean values are adjusted for age, smoking habits and predicted %FEV_1_

Table 5 reports chi-square results for dichotomized test outcomes stratified by sex. Significantly higher values were shown in exposed subjects for observed FEV_1_/FVC% <70% (in males) and for skin test ≥5 mm positivity (in females). Although it was quite weak and not statistically significant, a trend could also be highlighted for skin prick test ≥3 mm positivity in females and bronchial reactivity in males.

**Table 5:** Comparison of prevalence rates of tests variables by the distance classes in males and females.

	Males			Females		
	Distance of residence to main road			Distance of residence to main road		
	<100 m	100-250 m	250-800 m	<100 m	100-250 m	250-800 m
	%	%	%	%	%	%
Ige_log > 75^th^	34.0 (n = 191)	34.0 (n = 206)	29.6 (n = 291)	19.5 (n = 205)	16.3 (n = 208)	18.8 (n = 308)
Slope_ln > 75^th^	25.2 (n = 131)	22.4 (n = 152)	19.7 (n = 208)	27.3 (n = 88)	27.6 (n = 116)	30.5 (n = 164)
Skin test_5 mm pos.	18.7 (n = 209)	18.5 (n = 232)	17.5 (n = 331)	18.6 * (n = 226)	11.7 (n = 247)	11.8 (n = 363)
Skin test_3 mm pos.	34.9 (n = 209)	37.9 (n = 232)	34.4 (n = 331)	33.2 (n = 226)	28.7 (n = 247)	29.5 (n = 363)
FEV_1_/FVC% <70%	16.8 * (n = 191)	17.5 (n = 217)	9.6 (n = 303)	7.1 (n = 183)	5.6 (n = 214)	6.8 (n = 294)
FEV_1_/VC% <70%	14.7 (n = 191)	18.0 (n = 217)	12.9 (n = 303)	7.1 (n = 183)	3.7 (n = 214)	8.2 (n = 294)

*** p < 0.001, ** p < 0.01, * p < 0.05, # 0.05 < p < 0.1 (borderline); comparison between subjects living at different distances from the main road, separately in males and in females

Table 6 shows the statistically significant results (OR and 95% confidence intervals-CI) obtained from the multiple logistic regression models stratified by sex.

**Table 6:** Effects of distance of residence to main road on respiratory symptoms/diseases and dichotomized test outcomes: OR^† ^and 95% CI.

	Males		Females	
	<100 m	100-250 m	<100 m	100-250 m
Persistent wheeze	1.76 * (1.08-2.87)	1.54 # (0.94-2.53)	1.32 (0.76-2.28)	0.77 (0.42-1.42)
Dyspnea	0.88 (0.55-1.41)	0.86 (0.59-1.53)	1.61 ** (1.13-2.27)	1.35 # (0.95-1.93)
COPD	1.80 * (1.03-3.08)	1.21 (0.69-2.13)	1.60 (0.71-3.59)	0.99 (0.39-2.51)
Asthma	1.59 (0.85-2.98)	1.55 (0.83-2.87)	1.68 # (0.97-2.88)	0.58 (0.30-1.15)
Attacks of shortness of breath with wheeze	1.47 (0.87-2.48)	1.20 (0.70-2.04)	1.67 # (0.98-2.84)	0.74 (0.39-1.38)
Skin test_5 mm pos.	1.07 (0.67-1.72)	1.10 (0.70-1.73)	1.83 * (1.11-3.00)	0.95 (0.57-1.60)
FEV_1_/FVC% <70%	2.07 * (1.11-3.87)	2.53 ** (1.42-4.53)	1.01 (0.48-2.14)	0.88 (0.41-1.89)
FEV_1_/VC% <70%	1.15 (0.63-2.11)	1.76 * (1.02-3.04)	0.84 (0.40-1.72)	0.48 (0.21-1.11)

† OR adjusted for age, educational level, smoking habits, passive smoking exposure, occupational exposure, working position, number of hours spent at home and time of residence, calculated with subjects living between 250-800 m as the reference group

*** p < 0.001, ** p < 0.01, * p < 0.05, # 0.05 < p < 0.1 (borderline)

Compared to subjects living between 250 m and 800 m from the road, there were increased risks among males living within 100 m of the main road for persistent wheeze, COPD, and observed FEV_1_/FVC% < 70%; among males living between 100-250 m from the road, there were significantly increased risks for FEV_1_/FVC% < 70% and FEV_1_/VC% < 70%. A borderline significance was observed in men living between 100-250 m from the road for persistent wheeze. Increased risks were shown for dyspnea and for positivity to skin prick test ≥ 5 mm in females living within 100 m of the main road. Borderline effect estimates were observed for asthma, attacks of shortness of breath with wheeze in females living within 100 m of the road and for dyspnea in females living between 100-250 m from the road.

With regard to the estimated effects of potential confounders, our results confirmed what is well documented in the scientific literatures: risks for respiratory diseases were closely associated with age, smoking habits and low education levels (data not shown).

## Discussion

### Health issues

Our study indicates respiratory health risks for people living in the proximity of a main road. In our study we used subjects' residence as a proxy for environmental exposure. This means that subjects living at the same distance from the main road are assumed to experience the same exposure levels. Since personal exposure can be influenced by many different factors related to each subject's life style, personal habits and exposure to other air pollution sources, we included in our analyses the effects of these confounding factors. Multiple logistic regression models were fitted to adjust for the effects of age, education, smoking, passive smoking exposure, occupational exposure, working position, number of hours spent at home and time of residence.

After adjustment for such potential confounders, subjects with a residential address within 100 m of the main road had higher risks for reporting persistent wheeze, COPD, and for having airway obstruction in males, as well as higher risks for asthma, attacks of shortness of breath with wheezing, dyspnea and positivity to skin-prick test in females.

Our results are generally consistent with those reported by other authors who have analyzed the effects of traffic-related air pollution exposure on respiratory health status in adults.

Significantly higher risks for respiratory symptoms/diagnosis were reported in subjects living within 100 m of a major road by Lindgren et al. (Sweden) [37], Schikowski et al. (Germany) [12] and Cesaroni et al. (Italy) [38]: OR = 1.40 (95% CI 1.04-1.89) for asthma diagnosis, OR = 1.29 (95% CI 1.00-1.67) for asthma symptoms, OR = 1.64 (95% CI 1.11-2.41) for COPD diagnosis and OR = 1.53 (95% CI 1.10-2.13) for chronic bronchitis symptoms by Lindgren et al. [37]; OR = 1.24 (95% CI 1.03-1.49) for frequent cough by Schikowski et al. [12]; OR = 1.26 (95% CI 1.03-1.54) for rhinitis by Cesaroni et al. [38].

A narrower exposure cut-off (75 m) was defined by Mc Connell et al. [18] in Southern Californian schoolchildren (aged 5-7 years): significant associations were found for lifetime asthma (OR = 1.29 95% CI 1.01-1.86), current asthma (OR = 1.50 95% CI 1.16-1.95) and wheezes (OR = 1.40 95% CI 1.09-1.78). Effects of residential proximity to roadways were greater in females, as in our study.

Significantly higher risks for wheezes and phlegm were reported in adults living within 50 m of a major road by Garshick et al. (USA) [9] and within 20 m by Bayer-Oglesby et al. (Switzerland) [39]: OR = 1.30 (95% CI 1.00-1.70) for persistent wheezes and OR = 1.15 (95% CI 1.00-1.31) for regular phlegm, respectively.

In our study we also found elevated risks for airway obstruction in males living within 100 m of the main road, as well as between 100 m and 250 m from the road.

The study by Kan et al. [40] in the USA provided evidence that lung function, as measured by FEV_1 _and FVC, is reduced in adults living within 150 m of major roads, especially among women. In contrast to our results, they did not find a significant association between FEV_1_/FVC ratio and indicators of traffic exposure.

Adverse effects of traffic-related exposure on lung function have also been highlighted in other studies [41-43]. Gauderman et al. reported a reduced lung development in Californian children, with a not significant larger effect in boys than in girls [41]. Reduced lung function was reported by Forbes et al. [42] in English adults and by Abbey et al. [43] in Californian adults, with a larger effect in males.

With regard to the highest values of airway obstruction observed in the intermediate exposure class (100-250 m), this might be due to higher values for some confounding factors; although the estimates are adjusted for these factors, they still probably could have some residual influences. Furthermore, a few factors, unconsidered in the present analyses, might have influenced these results. For example, subjects living within 100-250 m of the road had an higher prevalence of childhood respiratory troubles (chest cold, pertussis and bronchitis) (data not shown); in a previous study we had shown that subjects with childhood respiratory troubles had the lowest lung function values regardless of smoking habits [44]. Anyway, the highest values of airway obstruction observed in the intermediate exposure class (100-250 m), might suggest that respiratory health impairments due to vehicular traffic exposure also occur at a distance greater than 100 m, as reported in other studies which have shown the negative effects of living near a busy road until a distance of 500 m [40,41].

As regards the atopic status, we found elevated risks for skin test ≥ 5 mm positivity in females living within 100 m of the main road.

A recent study on a very large sample of German children showed that the children living near busy streets had significantly higher risk for allergic sensitization (OR = 1.30, 95% CI 1.02-1.66) [45].

In the large population-based sample of 5338 schoolchildren of the French Six City Study [46] the adjusted odds of skin-prick test positivity were significantly higher than one in concurrence with elevated PM_2.5 _concentrations in the proximity of the houses where the children lived.

### Gender stratification

Although there is a growing epidemiological evidence of various associations between air pollution and respiratory health for males and females, few studies reported results stratified by gender in adults. Airway behaviour is influenced by sex-related (biological) and gender-related (socio-cultural) determinants; these aspects can interact to several degrees and directions with environmental exposures, differently in women and men. There may also be sex-based differences in susceptibility to the same environmental exposures [13]. These features can explain the different associations between sex and traffic air pollution found in our study.

Our approach focusing on the sex-specific effects pattern was also justified by the clear diversification between genders in the distribution of many confounding factors included in the analyses. Due to their prevalent occupation (housewives), females resulted more exposed to home residence environmental conditions; while men reported a greater risk for occupational and tobacco exposures.

### Advantages and disadvantages of study design

The main strengths of this study were the large sample size, the standard protocols, which already had passed the scrutiny of independent reviewers, and the multi-faceted aspects collected by means of the questionnaire. We also used quantitative respiratory and allergological outcomes (i.e. lung function, skin-prick test, IgE and bronchial hyper-responsiveness), which are not affected by the potential bias linked to the use of the questionnaire (recall bias).

As in any epidemiological study, residual confounding is still possible. However, we adjusted for known and potential confounders including demographic characteristics, personal socioeconomic status, lifestyle, work-related features, and cigarette smoking.

Another limitation was the cross-sectional nature of the study; we had no information about disease onset, making it hard to establish a temporal relationship between cause and effect. However, since asthma and COPD are known to be exacerbated by traffic-related air pollution, diseased subjects may have been more likely to move away from traffic, rather than towards it, and so a migrational bias would mainly be expected to dilute the effects.

We used a relatively simple proxy for exposure to traffic-related air pollution (distance to major roads): due to the large amount of input data required, we were not able to implement more sophisticated approaches; however, we found the distance method useful for an initial assessment of a potential environmental health hazard.

### Geographic issues

Address geocoding can also introduce bias and errors [47-50] with potential effects on the results of epidemiological studies [51-53].

Address matching can be hindered by several factors, such as incomplete or inaccurate information in the address files, lack of standardization of street addresses, and lack of assignment of house numbers, especially in rural areas [50-52]. We succeeded in matching almost 100% of our sample, after a considerable effort to overcome the above-mentioned problems.

Even if a match occurs, house numbering will not always provide the exact location since house numbers are assigned with no reference to the distance from the beginning of the street segment. Therefore, it is difficult to geocode an address unless the location of house numbers is identified on the map one by one.

Cartographic data provided by Pisa and Cascina municipalities contain the exact correspondence of house numbers to related buildings; a direct inspection was performed in case of ambiguity or uncertainty. Consequently, our study relied upon a detailed and precise geocoding of residential data, which is relatively infrequent for Italian public administrations.

## Conclusion

Our study points out that living in the proximity of the main road (within 100 m), as assessed by GIS technology, is associated with chronic respiratory problems, evaluated through both subjective (questionnaire) and objective (lung function and allergy tests) methods. In particular, living within 100 m of the main road was associated with higher risks for reporting persistent wheeze, COPD, and airway obstruction in males, as well as with higher risks for asthma, attacks of shortness of breath with wheezing, dyspnea and positivity to skin-prick tests in females.

In addition, our study highlights the added value of close collaboration among researchers with different expertise, such as epidemiology and geographical information system science, when conducting environmental epidemiology studies.

## List of abbreviations

GIS: Geographical information system; NO_x: _Nitrogen oxides; CO: Carbon monoxide; NMVOCs: Non-methane volatile organic compounds; LUR model: Land use regression model; COPD: Chronic obstructive pulmonary disease; DLCO: CO single breath diffusing capacity; FEV_1: _Forced expiratory volume in the first second; FVC: Forced vital capacity; VC: Vital capacity; OR: Odds ratio; 95% CI: 95% Confidence intervals.

## Competing interests

The authors declare that they have no competing interests.

## Authors' contributions

Each author contributed to the conception and design of the work, the acquisition of data, or the analysis of the data in a manner substantial enough to take public responsibility for it. The authors believe the manuscript represents valid work. All authors read and approved the final manuscript.
